# Solar Light-Induced Methylene Blue Removal over TiO_2_/AC Composites and Photocatalytic Regeneration

**DOI:** 10.3390/nano11113016

**Published:** 2021-11-10

**Authors:** Fernanda Dalto, Iwona Kuźniarska-Biernacka, Clara Pereira, Elsa Mesquita, Olívia Salomé G. P. Soares, M. Fernando R. Pereira, Maria João Rosa, Ana S. Mestre, Ana P. Carvalho, Cristina Freire

**Affiliations:** 1 REQUIMTE/LAQV, Departamento de Química e Bioquímica, Faculdade de Ciências, Universidade do Porto, Rua do Campo Alegre s/n, 4169-007 Porto, Portugal; up201309985@fc.up.pt (F.D.); iwonakb@fc.up.pt (I.K.-B.); clara.pereira@fc.up.pt (C.P.); 2 Water Quality and Treatment Laboratory, Urban Water Unit, Hydraulics and Environment Department, LNEC—National Laboratory for Civil Engineering, Av. Brasil 101, 1700-066 Lisboa, Portugal; emesquita@lnec.pt (E.M.); mjrosa@lnec.pt (M.J.R.); 3 LSRE-LCM, Departamento de Engenharia Química, Faculdade de Engenharia, Universidade do Porto, Rua Dr. Roberto Frias s/n, 4200-465 Porto, Portugal; osgps@fe.up.pt (O.S.G.P.S.); fpereira@fe.up.pt (M.F.R.P.); 4 Centro de Química Estrutural, Faculdade de Ciências, Universidade de Lisboa, Campo Grande, 1749-016 Lisboa, Portugal; ana.carvalho@fc.ul.pt

**Keywords:** titanium dioxide, activated carbon, nanocomposite, methylene blue, adsorption, solar-light induced photocatalysis, photoregeneration, wastewater, secondary effluent

## Abstract

TiO_2_-containing photocatalysts, which combine TiO_2_ with carbon-based materials, are promising materials for wastewater treatment due to synergistic photodegradation and adsorption phenomena. In this work, TiO_2_/AC composites were produced by the in situ immobilization of TiO_2_ nanoparticles over activated carbon (AC) derived from spent coffee grains, using different TiO_2_/AC proportions. The TiO_2_/AC composites were tested as adsorbents (dark) and as photocatalysts in a combined adsorption+photocatalytic process (solar irradiation) for methylene blue (MB) removal from ultrapure water, and from a secondary effluent (SecEf) of an urban wastewater treatment plant. All the materials were characterized by XRD (X-ray powder diffraction), N_2_ adsorption–desorption isotherms at −196 °C, SEM (scanning electron microscopy), UV-Vis diffuse reflectance, FTIR (Fourier-transform infrared spectroscopy), TPD (temperature programmed desorption), XPS (X-ray photoelectron spectroscopy) and TGA (thermogravimetric analysis). The TiAC60 (60% C) composite presented the lowest band gap (1.84 eV), while, for TiAC29 (29% C), the value was close to that of bare TiO_2_ (3.18 vs. 3.17 eV). Regardless of the material, the solar irradiation improved the percentage of MB discolouration when compared to adsorption in dark conditions. In the case of simultaneous adsorption+photocatalytic assays performed in ultrapure water, TiAC29 presented the fastest MB removal. Nevertheless, both TiAC29 and TiAC60 led to excellent MB removal percentages (96.1–98.1%). UV-induced photoregeneration was a promising strategy to recover the adsorption capacity of the materials, especially for TiAC60 and AC (>95%). When the assays were performed in SecEf, all the materials promoted discolouration percentages close to those obtained in ultrapure water. The bulk water parameters revealed that TiAC60 allowed the removal of a higher amount of MB, associated with the overall improvement of the SecEf quality.

## 1. Introduction

Synthetic dyes are widely used in many industries, such as food, plastics, textiles, etc. However, over 10–15% and 12% of the dyes are estimated to be lost during dyeing and manufacturing/processing operations, respectively. Approximately 20% of these lost dyes are released into industrial wastewater, causing environmental problems [1], since they may be toxic, carcinogenic, mutagenic, and hazardous to aquatic life, posing a threat to humans [2]. One such non-biodegradable dye, which is used in the textile and printing industry, is methylene blue (MB). Therefore, wastewater contaminated with dyes must be treated before discharge [3,4]. In recent years, photocatalysis is a featured and recurring theme in scientific research and sustainability studies. Within the context of wastewater treatment technologies, advanced oxidation processes (AOPs) based on the formation of hydroxyl radicals (•OH) and their use as primary oxidant for the degradation of organic pollutants deserve special attention [5]. Several alternative systems were developed to produce such radicals, namely photolysis, ozonation, Fenton and photo-Fenton, heterogeneous photocatalysis using semiconductors, and electro-assisted processes [6]. In particular, TiO_2_-based photocatalysis is a well-known technology for the removal of water pollutants mainly due to the large band gap of this oxide (anatase: 3.2 eV; rutile: 3.0 eV; brookite: 3.3 eV) [7,8] which, when irradiated with UV light (<385 nm), generates free radicals that are responsible for pollutant oxidation in water. Furthermore, TiO_2_ exhibits a high chemical stability, low cost and is non-toxic [9,10]. Among the TiO_2_-based photocatalysts, those working under solar light deserve special attention by particularly avoiding the extra consumption of energy and chemicals of wastewater treatments [11]. Several studies have proposed improvements in the catalytic activity of TiO_2_ by its doping with heteroatoms [12] or combination with magnetic nanoparticles [13]. Another strategy consists of the dispersion of the TiO_2_ catalyst onto porous supports in order to benefit from the synergistic effect of photodegradation and adsorption phenomena. In this case, zeolites, alumina, silica, carbon nanotubes, and activated carbon were used for dispersing TiO_2_ particles and improving their adsorption/degradation capacity [14,15,16,17,18,19].

Activated carbon (AC) appears as a remarkable support for TiO_2_ immobilization because it is a carbon-based material with a high surface area and relatively high porosity. AC is produced by the carbonization and activation of raw materials with a high carbon content and, preferentially, with a high density [20,21]. Several studies reported the production of ACs from waste materials, such as, fruit seeds [22,23,24], leather scraps [25], tires, cork [26,27], sisal [28,29] and coffee residues [30]. In the particular case of AC prepared by the activation of spent coffee grains, literature studies report large volume of pores and a high adsorption capacity for phenol [30] and H_2_S [31].

The combination of TiO_2_ with AC in a single composite material is a successful strategy to enhance the removal of dyes and other organic compounds from water and wastewater through the combined effect of adsorption and photodegradation [12,32]. The competitive performance of these composites mainly results from their improved photocatalytic activity, adsorption capacity, electron scavenging and sensitization ability, extended visible light absorption, and easier separation. The addition of AC to a semiconductor leads to various synergetic effects related to: (i) an increased quantity of substrate in contact with the immobilized metal oxide through adsorption [32], (ii) a better light harvesting due to the black character of the AC material [33], (iii) an improved charge separation in the presence of AC, and/or (iv) photosensitizing properties of the AC material [12].

The TiO_2_/AC composites (and other semiconductor/AC materials) can also promote the straightforward regeneration of the system by means of an oxidative process, instead of the ex situ and high-energy-consuming, conventional thermal regeneration. The oxidative regeneration through a photocatalytic process is a convenient but scarcely explored approach with potential to enhance both the removal and/or mineralization of the target pollutants and the number of reuse cycles without extra reagents consumption [34].

Therefore, the purpose of this study was to develop TiO_2_/AC catalysts for the removal of textile dye pollutants from water and wastewater. The AC was produced from the steam activation of spent coffee grains and then used as support for TiO_2_ particles that were formed in situ in the presence of the AC. The resulting TiO_2_/AC composites were tested as adsorbents and heterogeneous photocatalysts for the removal of MB dye (model pollutant) in an aqueous solution and in a wastewater effluent. Moreover, photo-regeneration assays were successfully performed.

## 2. Materials and Methods

### 2.1. Materials, Reagents and Solvents

Spent coffee grains (not including decaffeinated coffee) were collected from a local coffee shop and were dried until a constant weight was achieved. Titanium (IV) butoxide (97%) was acquired from Fluka (Buchs, Switzerland) and isopropanol (99%) was purchased from Analar Normapur (Leuven, Belgium). The concentrated HNO_3_ (65%) was obtained from Panreac (Barcelona, Spain), and potassium bromide (≥99%, FT−IR grade) was from Merck (Darmstadt, Germany). Ultra-pure water (Millipore, Darmstadt, Germany), specific resistivity 18 MΩ cm) was used throughout the experiments. All reagents were used without further purification. A sample of secondary effluent (SecEf) was collected from a Portuguese, conventional, urban wastewater treatment plant (WWTP). The organic matter content of the SecEf sample was assessed by measuring the dissolved organic carbon (DOC = 6.8 mgC/dm^3^) and UV-Vis absorbance at *λ* = 254 nm (A254 = 0.193 cm^−1^) and *λ* = 436 nm (A436 = 0.015 cm^−1^). The inorganic matrix was characterized by electrical conductivity (1.5 mS/cm), pH (7.5), and total hardness (284 mg CaCO_3_/L). SecEf turbidity was 2.3 NTU. These results were in the range of those reported for other urban WWTPs in Portugal [35] and in Spain [36] and show that this was a well-clarified and treated urban secondary effluent. This sample was filtered through 0.45 µm, polypropylene membrane (GH Polypro, Pall Corporation, Madrid, Spain) and then kept frozen until the assay day.

### 2.2. Preparation of the Activated Carbon

The AC used in the preparation of the TiO_2_/AC composites was obtained by steam activation of spent coffee grains. Briefly, around 32 g of dried biomass was introduced in a quartz reactor placed in a vertical furnace (Thermolyne, model 21100, Waltham, MA, USA), heated at 10 °C min^−1^ up to 900 °C under N_2_ flow (8 cm^3^ s^−1^). Once at that temperature, steam flow (generated in a bubbler half-filled with distilled water that was maintained at 90 °C and carried by the N_2_ flow) was introduced into the reactor and kept for 1 h, after which the sample was cooled down to room temperature under N_2_ flow. The burn-off of the process was 91%.

### 2.3. Preparation of the TiO_2_ Nanoparticles

The TiO_2_ nanoparticles were prepared by the sol-gel method following the procedure reported by Quiñones et al. [37]. The synthesis process started by diluting 4.2 cm^3^ of titanium(IV) butoxide into 1.4 cm^3^ of isopropanol. The resulting solution was slowly added to 30 cm^3^ of ultra-pure water acidified to pH 2 using concentrated HNO_3_. Afterwards, the resulting mixture was refluxed under stirring for 24 h. The obtained white precipitate was filtered, washed with ultra-pure water several times, and then washed with ethanol, until a neutral pH was achieved and dried at 100 °C overnight. Finally, the samples were heat-treated at 150, 200 or 250 °C in the absence of oxygen for 24 h.

### 2.4. Preparation of TiO_2_/AC Catalysts

The TiO_2_/AC composites were obtained by in situ precipitation of TiO_2_ in the presence of AC by the same method previously described in Section 2.3. The AC was dispersed in 30 cm^3^ of water acidified to pH 2 and sonicated for 5 min. The titanium (IV) butoxide solution in isopropanol was then added dropwise and the resulting mixture was refluxed under stirring for 24 h. The composites were recovered by filtration, washed with ultra-pure water and ethanol, and, afterwards, dried at 100 °C overnight and thermally treated at 200 °C under vacuum for 24 h. This temperature of heat treatment was selected, since, among all pure TiO_2_ nanomaterials prepared in Section 2.3, the material that was thermally treated at 200 °C presented the best photocatalytic performance (see Figure S1 in Supplementary Materials). Different amounts of titanium (IV) butoxide and AC were used to obtain TiO_2_:AC weight ratios of 50:50 and 10:90, assuming 100% yield in the synthesis of TiO_2_. The composites were designated by TiAC29 and TiAC60, i.e., through the general expression TiACX, where X represents the %C assessed by elemental analysis.

### 2.5. Methods

The carbon and nitrogen elemental analyses of AC and TiACX composites were obtained using an Elemental Analyzer apparatus (EA 1108 CHN-O Fisions Instruments, Waltham, MA, USA). The results are expressed in dry basis since the samples were previously dried at 105 °C. The Ti content present in the composites was analyzed by inductively coupled plasma (ICP) atomic emission spectroscopy (AES) using an ICP-AES Horiba Jobin-Yvon equipment, model Ultima (Piscataway, NJ, USA). All analyses were performed at “Laboratório de Análises”, Instituto Superior Técnico (Lisboa, Portugal).

Powder X-ray diffraction (XRD) was carried out on an X-ray Phillips PX 1730 diffractometer (Philips, Eindhoven, Netherlands) with automatic data acquisition (APD Philips (v3.6B) software (Philips, Eindhoven, The Netherlands), using Cu Kα (*λ* = 1.5406 Å) radiation as incident beam (40 kV; 30 mA). The diffractograms were obtained by continuous scanning from 2*θ* = 20° to 80°, with a step size of 0.015° and a time per step of 0.4 s.

The XPS (X-ray photoelectron spectroscopy) analysis was performed on a Kratos AXIS Ultra HSA (Manchester, UK), with VISION software for data acquisition, at a pressure lower than 1 × 10^−6^ Pa. The analysis was carried out with a monochromatic Al Kα X-ray source (1486.7 eV), operating at 15 kV (90 W), in FAT mode (Fixed Analyzer Transmission), and using a charge neutralization system. For the survey spectra acquisition, a pass energy of 80 eV, a step of 1 eV and a dwell time of 100 ms were used. The effect of the electric charge was corrected by the reference of the C 1s peak at 285.0 eV.

The textural characterization of the AC material and TiACX composites was made by N_2_ adsorption–desorption isotherms at −196 °C on an automatic apparatus model, ASAP 2010, from Micromeritics (Norcross, GA, USA). Before the experiments, the samples (~60 mg) were outgassed overnight at 120 °C, under vacuum better than 10^−2^ Pa. The nitrogen adsorption data were used to estimate the apparent surface area, *S*_BET_, evaluated through BET equation [36,38], which was applied in the *p/p*^0^ range determined following the methodology reported in ISO-9277 [39]. The micropore volume (*V*_micro_) was determined using the *t*-method. The mesopore volume (*V*_meso_) was determined through the difference between the total pore volume (*V*_total_, corresponding to the volume of N_2_ adsorbed at *p/p*^0^ = 0.975) and *V*_micro_.

Fourier transform infrared (FTIR) spectra were recorded on a Jasco FTIR spectrometer, model FT/IR-460 (Jasco, Tokyo, Japan), in the wavenumber range of 4000–400 cm^−1^, with a resolution of 4 cm^−1^ and 32 scans. The spectra were obtained in KBr pellets containing 1 wt.% of the material.

UV-Vis diffuse reflectance spectra (*λ* = 220–600 nm) of the powder samples were recorded on a Jasco V-560 UV-Vis spectrophotometer (Tokyo, Japan), with a double-monochromator, double-beam optical system. The spectrophotometer was equipped with an integrating sphere attachment (Jasco ISV-469, Tokyo, Japan). The reflectance spectra were converted to equivalent absorption Kubelka–Munk units by the instrument software (Jasco, Tokyo, Japan).

The surface chemistry was characterized by temperature-programmed desorption (TPD) analysis. The CO and CO_2_ TPD spectra were obtained on a fully automated AMI 300 Catalyst apparatus (Altamira Instruments, Cumming, CA, USA) connected to a Dycor Dymaxion Mass spectrometer (Meerbusch, Germany). Each sample (70.0 mg) was heated up to 1100 °C at 5 °C min^−1^ using a constant flow rate of helium equal to 25 cm^3^ min^−1^. For quantification of the CO and CO_2_ released during the analysis, calibration of these gases was carried out at the end of each experiment.

Thermogravimetric (TG) analysis was carried out under air, with a heating ramp of 10 °C min^−1^ from 50 to 900 °C, on an STA 409 PC/4/H Luxx Netzsch thermal analyzer (Selb, Germany).

The morphology of the samples was characterized by scanning electron microscopy (SEM, Hillsboro, OR, USA). The micrographs were obtained at CEMUP (Materials Centre of the University of Porto, Porto, Portugal) using a FEI Quanta 400 FEG microscope, working at an accelerating voltage of 15 kV, and attached to an EDAX Genesis X4M X-ray spectrometer (Hillsboro, OR, USA). Transmission electron microscopy (TEM) characterization of the TiO_2_ nanoparticles was performed on a Hitachi H8100 microscope (Krefeld, Germany).

### 2.6. Adsorption and Photocatalytic Performance Tests

The adsorption and photocatalytic properties of the obtained materials were evaluated using MB as model pollutant on a sunlight simulator equipment Q-SUN Xe-1 Xenon Test Chamber Model LX-5060 (Zhangzhou, China) (full spectrum sunlight). For these assays, the TiO_2_ sample calcined at 200 °C was tested along with the AC and the TiACX composites.

Ultraviolet-visible (UV-Vis) spectra of aqueous MB solutions were acquired on a Varian Cary50 Bio spectrophotometer (Santa Clara, CA, USA), in the range of *λ* = 200–800 nm. The concentration of the MB dye was calculated by a standard curve at the maximum absorption of “pure” MB in water (*λ =* 665 nm).

Adsorption assays were made in the dark (without light irradiation). Briefly, 20 cm^3^ of MB solution (25 mg dm^−3^) and 10 mg of catalyst were introduced in a 50 cm^3^ reactor, placed on the sunlight simulator (solar box) and stirred at 200 rpm for 90 min at room temperature in the dark. The samples were collected at fixed time intervals, centrifuged and analyzed by UV-Vis spectroscopy (0.20 cm^3^ aliquots were diluted when necessary).

The amount of MB adsorbed per gram of adsorbent, *q_f_* (mg g^−1^), was calculated according to the following equation:

(1) $$q_{f}=\frac{V\left(C_{0}-C_{f}\right)}{W}$$

where *C*_0_ and *C_f_* are the initial and final (after 90 min of contact) concentrations of MB (mg dm^−3^), respectively, *V* is the volume of the solution (dm^3^), and *W* is the mass of adsorbent (g).

The MB dye removal percentage (%*R*), also denominated as discolouration percentage, was calculated as follows:

(2) $$\%R=\frac{\left(A_{0}-A_{t}\right)}{A_{0}}\times 100$$

where *A*_0_ and *A_t_* are the absorbance values corresponding to *λ*_max_ = 665 nm at the beginning of the absorption process and at any time, respectively.

The photocatalytic assays were performed under similar experimental conditions to those used in the adsorption tests, i.e., without any previous equilibration steps under dark conditions. The assays were performed in the same solar box under simulated full spectrum sunlight irradiation (i.e., *λ* > 420 nm, 0.8 W m^−2^). A blank experiment (photolysis) was performed in the absence of the catalysts under light irradiation. Both adsorption and photocatalytic tests were performed for up to 90 min to minimize the temperature effect on photocatalytic and adsorption properties of materials tested.

For the reuse tests, the materials used in a first photocatalytic cycle were recovered by filtration, washed with water and ethanol, dried under vacuum, and tested under the same experimental conditions, by adjusting the volume of MB solution to the available material.

In the case of regeneration + reuse assays, the recovered catalysts (separated by filtration after the first photocatalytic cycle) were suspended in 200 cm^3^ of ultrapure water and irradiated by UV light (high-pressure Hg lamp, 400 W) for 60 min. After the irradiation process, the materials were separated by filtration, washed with water and ethanol, and dried under vacuum overnight. The dried samples were used as catalysts in the next catalytic cycle for simultaneous adsorption and discolouration of MB under simulated solar light irradiation.

The experiments using WWTP secondary effluent as water matrix were performed under the same operating conditions (amount of catalyst and MB concentration) as those used in the adsorption and catalytic tests in ultrapure water. After the experiments, the materials were filtered from the solutions with a 0.45 µm nylon syringe filter.

The organic matter contents of the SecEf sample and of the samples from the adsorption and photodegradation tests performed in SecEf were assessed by measuring the dissolved organic carbon (DOC) and UV-Vis absorbance. The DOC was determined in filtered samples (0.45 µm, polypropylene membrane, GH Polypro Pall Corporation) by the UV/persulphate chemical oxidation method [40] using a TOC analyzer (Teledyne, TOC Fusion, Thousand Oaks, CA, USA). The UV-Vis absorbance values of the filtered samples (as for DOC) at *λ* = 254 nm (A254) and *λ* = 436 nm (A436) were measured, corresponding to organic compounds with aromatic rings and color, respectively (UV/Vis spectrophotometer, Thermo Scientific—Evolution 201 (Waltham, MA, USA), on 10 mm optical path length quartz cells). The pH and EC of SecEf were measured with a multiparametric potentiometer (Consort, C863T) and total hardness was determined by volumetry using standard methods for wastewater analysis [41].

## 3. Results and Discussion

### 3.1. Characterization of Materials

The AC and TiACX composites were characterized by chemical analyses to directly determine the total amount of carbon, nitrogen, and titanium (Table 1). In the case of the composites, as expected, the Ti loadings of TiAC29 and TiAC60 composites were 22.6% and 5.6%, respectively, while the %C values were 29.0 and 60.2%.

**Figure 1:**
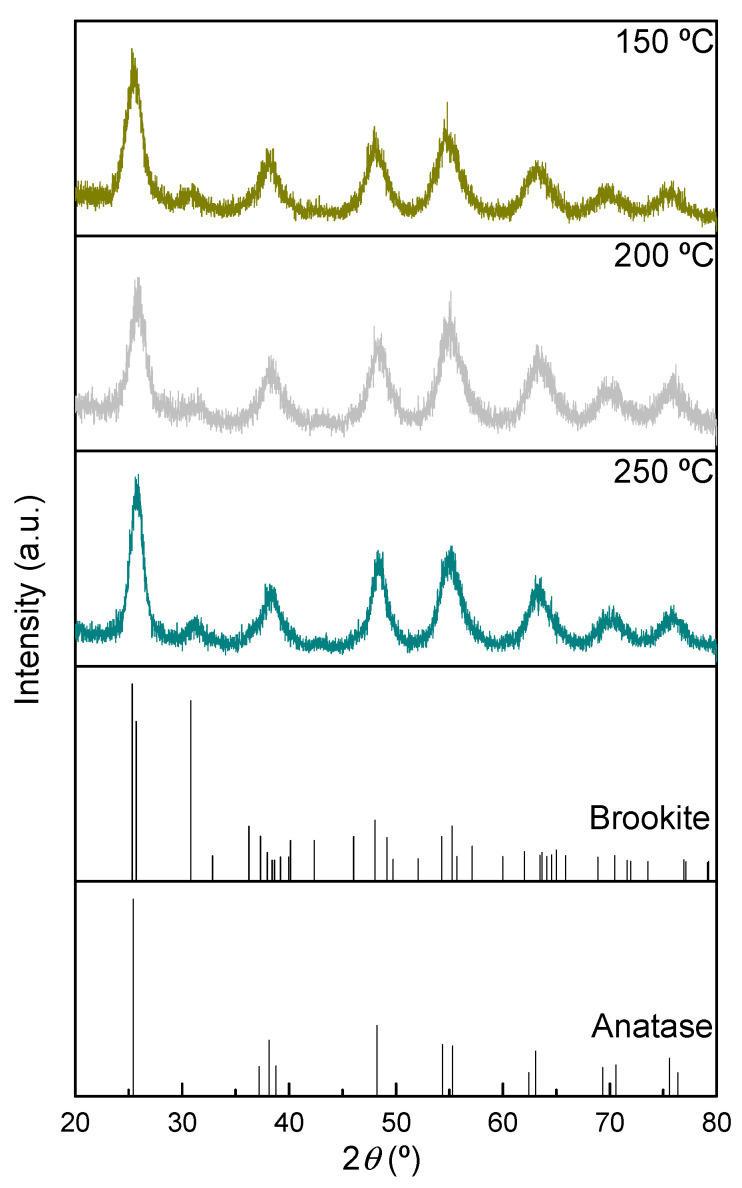
XRD patterns of TiO_2_ samples thermally treated under vacuum at different temperatures. Patterns used as standards for pure anatase and brookite samples from Crystallography Open Database (COD ID: 1526931 and 9009087, respectively).

The XRD patterns of the TiO_2_ samples prepared ex situ (Figure 1) show that, in all cases, a mixture of anatase, with a tetragonal structure (space group: *I*4_1_/*amd*, COD ID: 1526931) and brookite, with an orthorhombic structure (space group: *Pbca*, COD ID: 9009087) is present [42,43,44,45], with anatase being the dominant phase. The overlap of the diffraction peaks corresponding to the two phases in all the diffractograms, along with the broadening of the peaks, hinder the determination of the crystallite size associated with each phase.

On the other hand, the increase in the temperature of the thermal treatment during the preparation of the samples does not lead to relevant changes in the corresponding XRD patterns, indicating that the types of phases are preserved. The observed peak broadening in all diffractograms indicates the presence of small crystallite domains.

**Figure 2:**
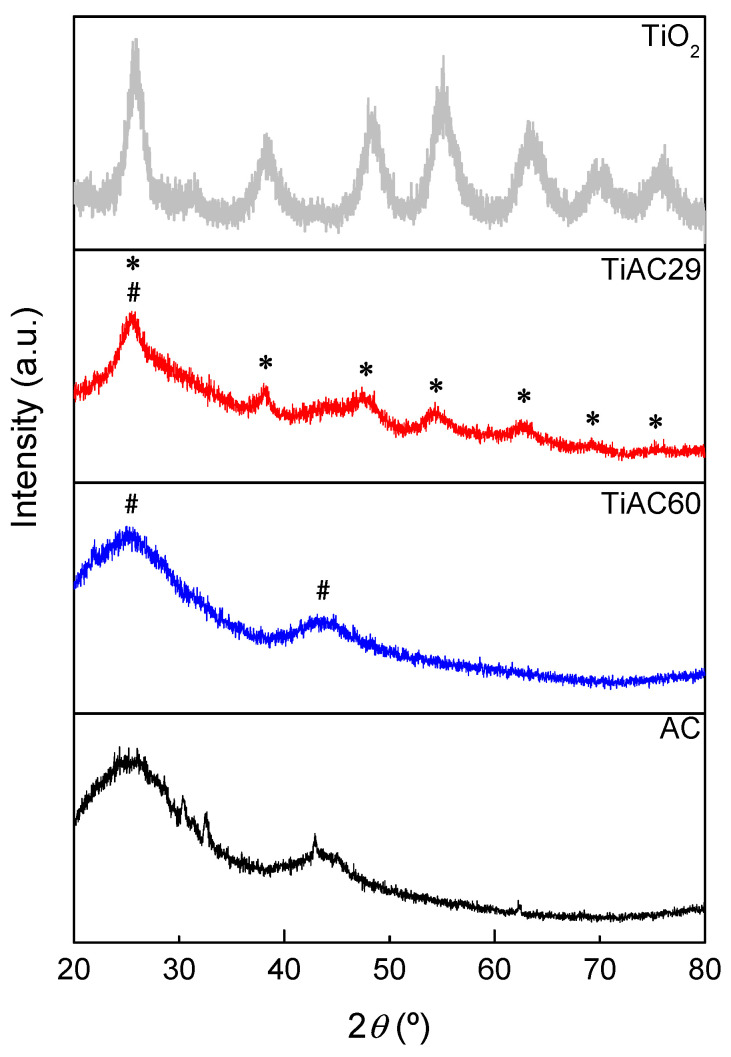
XRD patterns of the AC, TiO_2_ and TiACX composites (#—characteristic peaks of AC component; *—peaks assigned to TiO_2_ component within the composites).

The XRD patterns of TiO_2_ calcined at 200 °C, activated carbon, and TiACX composites are displayed in Figure 2. The XRD pattern of the AC derived from spent coffee grains displays two broad bands at around 2*θ* = 25.5° and 44.0° [ICDD 25-284], which are characteristic of (002) and (100) reflections from an amorphous-like carbon structure (highlighted with “#” symbol in Figure 2) [29]. These two bands are the only reflections detected in the diffractogram of the TiAC60 composite due to the considerable amount of carbon component present in this sample. The TiAC29 sample, with 4.0× higher Ti loading and 2.1× lower carbon content than that of TiAC60, shows a different pattern with several additional peaks assigned to the TiO2 component (highlighted with “*” symbol in Figure 2).

**Figure 3:**
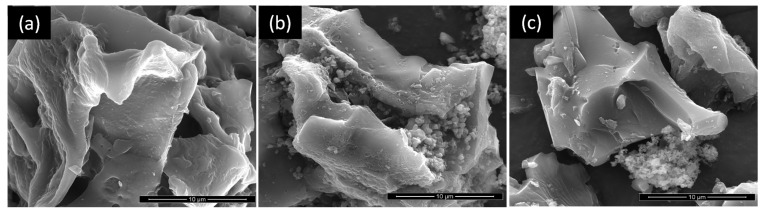
SEM images of (**a**) AC, (**b**), TiAC29, and (**c**) TiAC60.

The TEM image of TiO_2_ calcined at 200 °C (Figure S2 of Supplementary Materials) reveals that it is composed of nanoparticles with dimensions around 5–6 nm. The morphology of the parent AC and TiACX composites was evaluated by SEM, and the corresponding micrographs are presented in Figure 3. The AC presents a sheet-like morphology that is preserved in the TiACX composites. The micrographs of TiAC29 and TiAC60 reveal a non-uniform distribution of the TiO_2_ particles on the composites, with a higher degree of aggregation close to the carbon surface.

**Figure 4:**
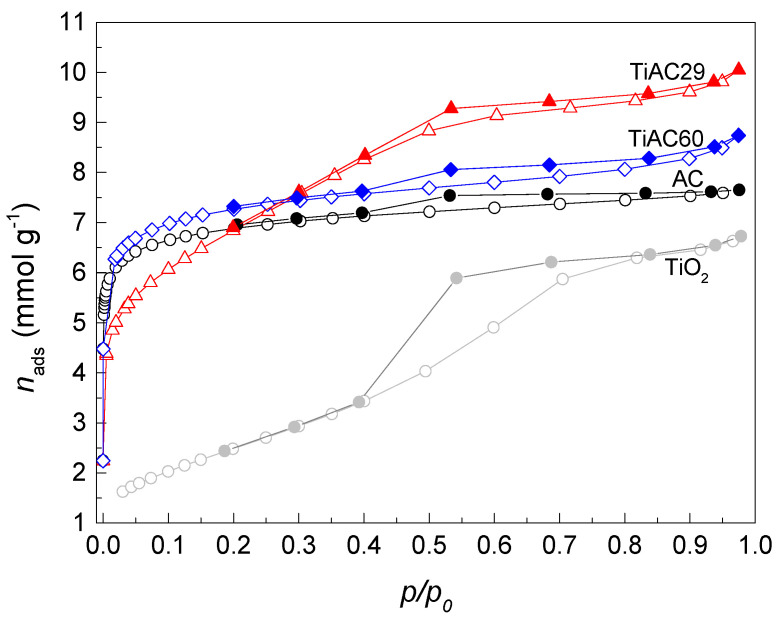
N_2_ adsorption–desorption isotherms at −196 °C of the AC, TiO_2_ and TiACX composites. The empty and filled symbols represent adsorption and desorption data, respectively.

The N_2_ adsorption–desorption isotherms at −196 °C are presented in Figure 4 and show the different textural characteristics of the samples. The AC isotherm is of type I + IV, revealing the essential microporous nature of the sample, with a H4 hysteresis loop, which is characteristic of slit-shaped mesopores in the activated carbon materials. In the case of TiO_2_, a type IV(a) isotherm with a H2(b) hysteresis loop was obtained, indicating the existence of mesopores within the agglomerates of spherical particles [46], typical of the TiO_2_ particles observed in Figure S2 of the Supplementary Materials. The configuration of the isotherm obtained for the TiAC60 composite is similar to the curve of AC. In the case of the isotherm of TiAC29 composite, the mesoporous character is dominant, as revealed by the step adsorption up to the relative pressure values around 0.6, which is likely due to the high percentage of the oxide in the composition of this material. The large amount of TiO_2_ particles present in TiAC29, and their location on the interstitial spaces of AC, are also responsible for the slight decrease in the BET surface area and micropore volume (Table 2). This is the composite with textural properties that resembles those of both components, presenting a well-developed micro+mesoporous structure.

**Figure 5:**
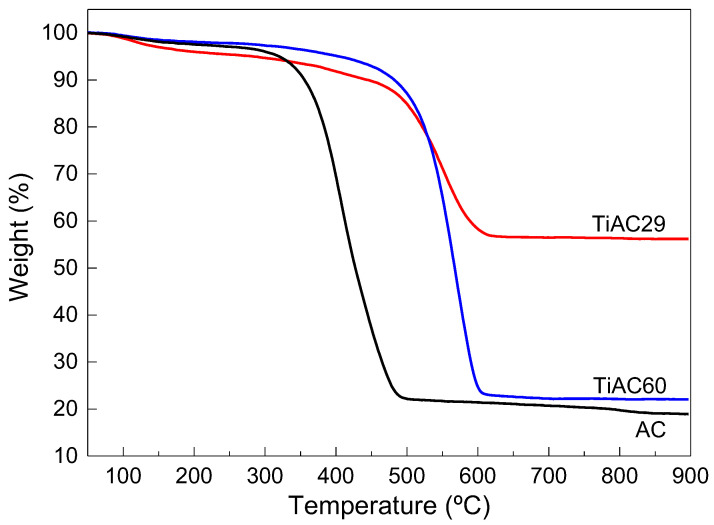
Thermogravimetric curves of bare AC and TiACX composites obtained under air flow.

The thermograms of the composites and AC are displayed in Figure 5. For all samples, there is a minor mass loss in the range of 50–150 °C associated with water removal. Afterwards, there is a more significant weight loss that occurs in two stages: in the first stage, between 150 °C and 280 °C, which can be associated with the removal of more labile surface groups (e.g., carboxylic groups), there is no relevant weight loss [47]. The second and more pronounced weight loss occurs between 350 °C and 500 °C for the AC and between 500 °C and 600 °C for the composites, related to the decomposition of the other remaining surface functionalities and carbon structure degradation. In this sense, the presence of TiO_2_ in the composites promotes a shift in the decomposition temperature to higher values.

The ash content of the AC was estimated from the residue of the assay, representing 19% of the sample weight. Regarding the composites, as expected, the higher the %Ti, the lower the total weight loss and, consequently, the higher the amount of solid residue at the end of the assay. The XPS spectra of AC (Figure S3 of Supplementary Materials) reveals that, as expected, C and O are the main elements at the AC surface with atomic percentages of 82.72% and 14.83%, respectively. Lower percentages of N and Si were also detected. Although Si accounts for 1.55 At%, it must be noted that this quantification was made using a tape that contained an unknown amount of Si, as well as unknown amounts of C and O. A trace amount of sulfur was detected in AC, which could be due to contaminants on the spent coffee grains. Therefore, we can conclude that the AC material is relatively pure and does not contain metal oxides that can be relevant for the target application.

**Figure 6:**
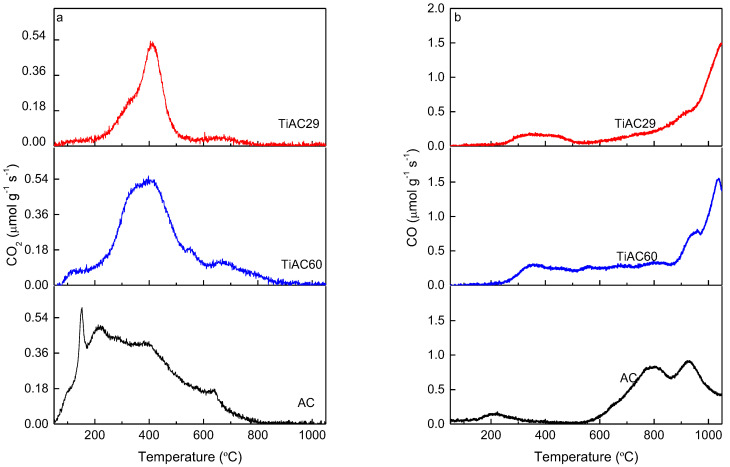
TPD profiles of AC and TiACX composites: (**a**) CO_2_ evolution; (**b**) CO evolution.

TPD analysis allows the identification of the different types of oxygen-based groups on the carbon surface. With this technique, the surface groups are decomposed at different temperatures upon heating the material, with CO, CO_2_ and H_2_O being the main released compounds. Figure 6 shows the TPD profiles of the released CO and CO_2_ obtained upon the heating of AC and the composites. The amounts of CO and CO_2_ released from the samples surface, quoted in Table 3, were obtained by the integration of the areas under the corresponding TPD profiles. The CO_2_ evolution profile for the sample AC mainly results from the decomposition of carboxylic acid groups, since these groups are decomposed at lower temperatures (from 100 °C to 450 °C) [47,48]. This profile also shows bands at temperatures between 400 °C and 650 °C associated with the decomposition of anhydrides and lactones, respectively, which are more stable than the carboxylic groups [47]. The CO evolution profile reveals that the highest amount of CO is released in the range of 600–1000 °C, which indicates the presence of mainly carbonyl and quinone groups that decompose at a temperature around 800 °C [47].

The results presented in Table 3 show that all samples have a higher amount of surface groups decomposing into CO than into CO_2_, pointing out the basic characteristic of these materials. The CO_2_/CO ratios are lower for the composites than for the AC material, confirming that the reaction conditions used during their synthesis promoted the increase in their basic character to a larger extent for TiAC29, the Ti-richest composite.

**Figure 7:**
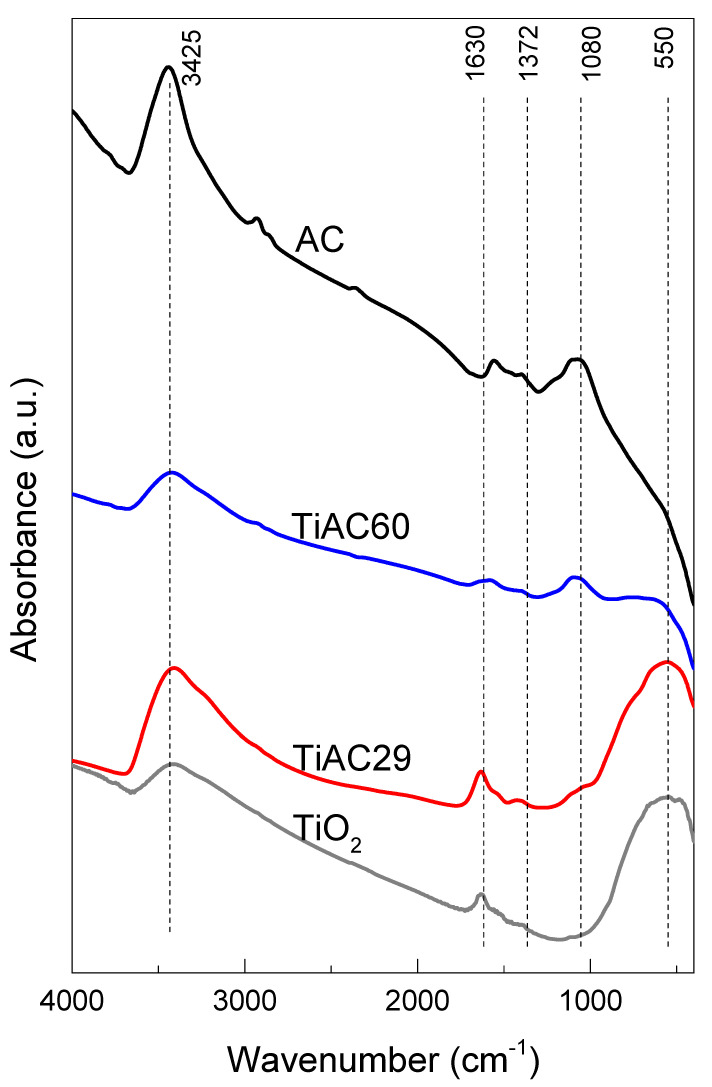
FTIR spectra of AC, TiO_2,_ and TiACX composites.

The FTIR spectra of the studied materials are presented in Figure 7.

The FTIR spectrum of the parent TiO_2_ presents the characteristic broad band related to the titanium dioxide framework, centred around 550 cm^−1^, which is attributed to the stretching vibrations of Ti–O–Ti. A broad band is detected around 3425 cm^−1^, which is assigned to the stretching vibrations of the O–H groups from the TiO_2_ surface and adsorbed water, while the band at 1630 cm^−1^ corresponds to the O–H bending vibration from adsorbed water [49]. No bands associated with titanium (IV) butoxide (expected at 1458, 1429, and 1362 cm^−1^) were detected in the spectra of all TiO_2_-based samples [50], showing that the calcination step at 200 °C led to the full hydrolysis and condensation of the precursor into TiO_2_.

The FTIR spectrum of AC (Figure 7) shows a weak band at around 2930 cm^−1^ corresponding to symmetric and asymmetric C–H stretching vibrations of aliphatic moieties [51], and a band at 1560 cm^−1^ assigned to C=C stretching modes in aromatic rings [52]. The existence of aliphatic moieties is also confirmed by the presence of a weak band located at ca. 1370 cm^−1^ due to C–H bending vibrations [48]. The more intense band, centered at 1080 cm^−1^, is attributed to C–O stretching vibrations in the ether or phenol groups [52]. The spectrum also presents a broad band at 3425 cm^−1^ due to the stretching vibrations of O–H in the surface hydroxylic groups and/or from adsorbed water [51,52].

The FTIR spectra of the TiACX composites present the characteristic bands of AC as well as of TiO_2_, confirming the successful preparation of the composites. The intensity of the band associated with TiO_2_ (550 cm^−1^) in the spectra of the composites is, as expected, more intense for the TiAC29 composite, being in accordance with the results obtained by the elemental analyses. Concomitantly, the intensity of the characteristic band of AC at 1080 cm^−1^ is higher for TiAC60, in line with its higher %C. It can also be noticed that the band is slightly shifted to higher wavenumbers in the spectra of the composites, which suggests the existence of interactions between the AC and TiO_2_ components.

**Figure 8:**
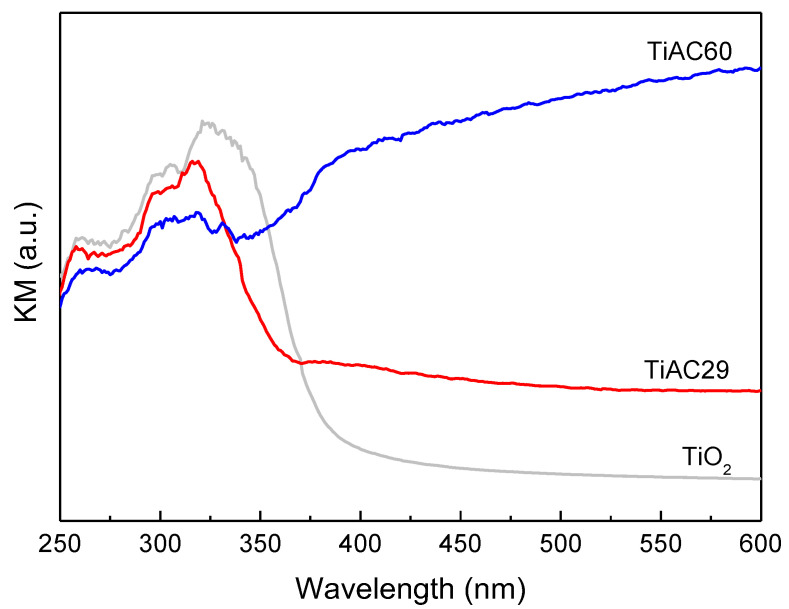
Diffuse reflectance spectra of bare TiO_2_ and composites.

The diffuse reflectance spectra of the composites are displayed in Figure 8. The spectrum of the bare TiO_2_ only reveals the existence of absorption in the UV region, whereas the presence of the AC component in the composites promotes an increase in the absorption pattern in the visible range (*λ* > 400 nm) due to the black characteristic of AC. In the UV range, the spectra of the composites show the typical profile of TiO_2_ but with a blue shift and narrower absorption band when compared with the spectrum of pure TiO_2_.

The band gap energy values for bare TiO_2_ and TiACX composites were determined from the Tauc plot [53], where the linear part of the plot was extrapolated to the x-axis. The band gap energies are 3.17, 3.18 and 1.84 eV for TiO_2_, TiAC29 and TiAC60, respectively. Thus, it can be inferred that the band gap of TiO_2_ becomes considerably narrower when combined with AC, which is in accordance with the literature reports on TiO_2_/carbon materials (e.g., C-doping, carbon nanotubes, graphene oxide, and carbon quantum dots) [12].

### 3.2. Evaluation of Adsorption and Photocatalytic Activity of TiAC Composites over Simulated Solar Light

#### 3.2.1. Adsorption Studies

**Figure 9:**
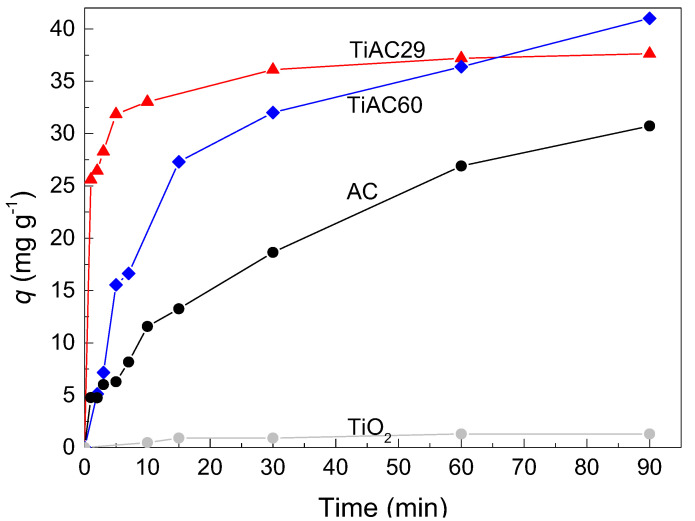
Effect of contact time on MB dye adsorption in the dark for bare materials and composites (dosage = 0.5 g dm^−3^, V = 0.02 dm^3^, [MB] = 25 mg dm^−3^).

MB adsorption studies were performed for the parent AC and TiO_2_ and for the TiACX composites. The effect of the contact time on the MB adsorption by the different samples is shown in Figure 9. As expected, the MB uptake by TiO_2_ is practically negligible (resulting in MB removal percentage at 90 min of contact time, %R < 5%); on the contrary, AC shows a significant MB adsorption (higher *q* and %R = 68% after 90 min of contact time). For the TiO_2_ and TiAC29 composites, the contact time of 90 min is enough for the adsorption equilibrium, which is not the case for the AC and TiAC60 composites. In fact, after the initial steep increase in MB uptake, a relatively pronounced slope is observed for these systems. In the case of TiAC60, it must also be noticed that, aside from its different adsorption profile when compared to that of TiAC29, it shows another unexpected behaviour, since, after 90 min of contact time, it presents the highest MB uptake.

To understand this behaviour, we must bear in mind that the MB adsorption mechanism can be controlled by various processes: electrostatic interactions, π–π interactions, and hydrogen bonds. From the set of tested materials, AC presents the most acidic surface chemistry (see TPD discussion). Therefore, it is likely that both electrostatic interactions between the cationic dye and a negatively-charged carbon surface, and π–π interactions between the π electrons of the aromatic structure of MB and the delocalized π electrons on the basal planes of the bare AC and AC fraction of the composites, are responsible for the adsorption mechanism [54,55]. Regarding the TiACX composites, the TPD data clearly show a more basic surface that hinders the occurrence of electrostatic interactions. Hence, in these systems, the adsorption mechanism seems to have a major contribution from hydrogen bonds between the amine moieties of the MB and -OH groups (from AC but also from TiO_2_) and/or with the nitrogen-containing surface groups in AC and TiACX composites (see %N Table 1). In fact, the relatively intense bands at 3425 and 1630 cm^−1^ in the FTIR spectrum of the TiAC29 sample, which correspond to O–H stretching and bending vibrations, may justify the faster adsorption rate for this composite. Thus, it seems that the contribution of hydrogen bonds is more relevant for the composites as the %Ti increases. In turn, the bare TiO_2_ presents an almost negligible MB removal. These findings support the existence of the synergetic effect related to the confinement of MB in the adsorption active sites, favouring the contact with the immobilized metal oxide.

**Figure 10:**
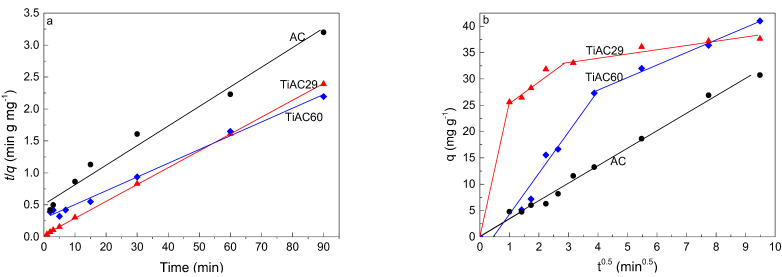
Pseudo-second-order kinetics plots (**a**) for the adsorption of MB onto bare AC and composites, and (**b**) Weber and Morris intra-particle diffusion plots (dosage = 0.5 g dm^−3^, V = 0.02 dm^3^, [MB] = 25 mg dm^−3^).

To evaluate the kinetic mechanism that controls the adsorption process, the kinetic data were analyzed using four kinetic models, including the Lagergren first-order [56], the McKay and Ho pseudo-second-order [57], Weber−Morris intraparticle diffusion [58], and Elovich models [59,60] (equations presented in the Supplementary Materials). Due to the very low adsorption of MB by bare TiO_2_, this system was not analyzed. Table 4 shows the parameters obtained from the fits of the adsorption kinetic models for AC and TiACX samples. For all samples, the correlation coefficients (R^2^) from all fits were greater than 0.80, with the highest values being observed for the fits with the pseudo-second-order kinetic model. The applicability of the pseudo-second-order kinetic model (Figure 10a) supports the fact that the *q*_f_ values obtained by the fitting agree with the *q*_f_^exp^ values. This suggests that the rate of the adsorption process is preferably controlled by chemisorption, as anticipated in previous discussion, which is in agreement with reports presented by other authors on MB dye adsorption over NaOH-activated carbon produced from coconut shells [61], or activated carbons from waste biomass [62].

The experimental data fit well in the Elovich equation with high values of correlation coefficients (R^2^ from 0.92 to 0.99), Figure S4 (Supplementary Materials) and Table 4. This model is successfully applied for reactions involving the chemisorption of adsorbate on a solid surface without the desorption of products. Accordingly, it can be concluded that the type of interaction of the MB with the adsorbents is chemisorption. Similar results were reported in the adsorption of MB onto carbon nanotubes (CNTs) [63], activated carbon prepared from date press cake [64], and biomass-derived activated carbon [65].

To better understand the diffusion rate controlling procedure, the intra-particle diffusion model was applied (Figure 10b). This model assumes (i) the direction of diffusion is radial; (ii) the external resistance to the mass transfer of adsorbate molecules is not significant, or only significant within a short time at the beginning of diffusion; and (iii) the intraparticle (pore) diffusivity does not change with time nor with position [64,66]. Based on these assumptions, the diffusion process may occur in three stages: (i) the mass transfer from the bulk solution to the external surface of the adsorbent by diffusion through the liquid film layer, (ii) mass transfer to the pores of the adsorbent, and (iii) adsorption of adsorbate molecules onto the internal surface of the pores [64,67].

As shown in Figure 10b, the whole MB adsorption profile shows three linear regions for TiAC29 (three slopes) and two linear regions for TiAC60, suggesting that multiple steps occur during the adsorption process. The piecewise fitting parameters of the intra-particle diffusion are listed in Table 4. The first stage, starting from the origin, is related to the transfer of adsorbate molecules from bulk liquid to the external surface of the adsorbent. The second stage is the gradual sorption process, where intraparticle diffusion is rate controlled, i.e., the entrance of the adsorbate molecules into the porous structure of composites. The third stage (which only exists for TiAC29, as expected, since it is the only material whose profile reaches equilibrium), with a small slope, is the final equilibrium stage, where intraparticle diffusion starts to slow down due to a low solute concentration in the solution [68,69]. The conclusion from the abovementioned observation is that there are three (for TiAC29) or two (for TiAC60) different rate-determining steps that switched from one to the other(s) as the adsorption process proceeded. However, from the results, it is not possible to say which of these processes is the overall rate-limiting step of the adsorption mechanism.

In conclusion, the overall kinetic mechanism seems to be more related to surface functionalities than to the textural properties of the materials since, for example, AC and TiAC60 have similar micro-mesopore networks, but the latter presents a faster MB adsorption profile and higher MB uptake.

#### 3.2.2. Combined Adsorption and Photocatalytic Process

**Figure 11:**
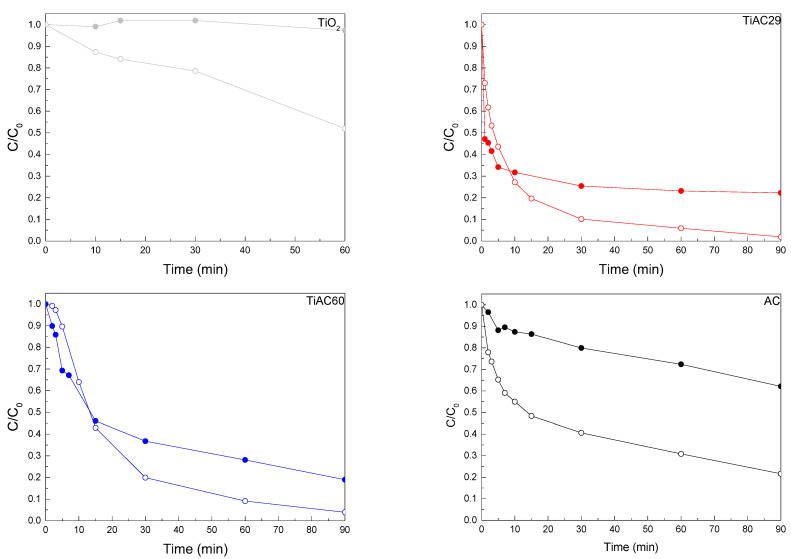
Comparison of MB adsorption (filled symbols) and solution discolouration by combined adsorption+photocatalysis under simulated solar light irradiation (open symbols) over bare materials and composites.

The kinetic adsorption profiles (dark conditions) and combined adsorption+photocatalysis profiles under simulated solar light irradiation are presented in Figure 11. During the blank experiment (i.e., photolysis) the MB solution was discoloured very slowly under simulated solar light irradiation (less than 5% during 180 min irradiation; see Figure S5 in Supplementary Materials). The AC, TiO_2_, and the composites adsorbed up to 81% of MB dye during 90 min of contact, thus the adsorption+photocatalytic behaviour (under simulated solar irradiation) of the materials was simultaneously studied.

The comparison between MB concentration variation during adsorption (dark experiment) and simultaneous adsorption + photocatalysis (light irradiation) shows that, even though adsorption played an important role in the discolouring process for all the samples, the presence of the visible light had a positive effect on MB discolouration (Figure 11 and Table 5). The presence of AC in the composites increased the photoactivity of TiO_2_, most likely because, as previously discussed, the high surface area of AC effectively concentrated MB around the deposited TiO_2_. This improved removal via the bare AC under adsorption + photocatalytic conditions is in line with the previously reported results from Velasco et al. [70], showing that nanoporous carbons can exhibit visible-light, photocatalytic activity in the case of phenol photooxidation. The adsorption and photocatalytic removal of MB under UV irradiation was also addressed by Atout et al., where TiO_2_ was supported on a commercial granular-activated carbon [71]. Although the granular-activated carbon material presented a nanoporous network similar to that of the herein developed activated carbon, in their study, the carbon material did not lead to the UV photocatalytic degradation of MB; after 1 h of UV irradiation, the composite containing TiO_2_ only led to a 45% MB removal, while, in the present work, after 1 h of solar irradiation, our AC promoted a 70% MB removal, and the TiAC29 and TiAC60 composites reached ≈90% MB removal.

**Figure 12:**
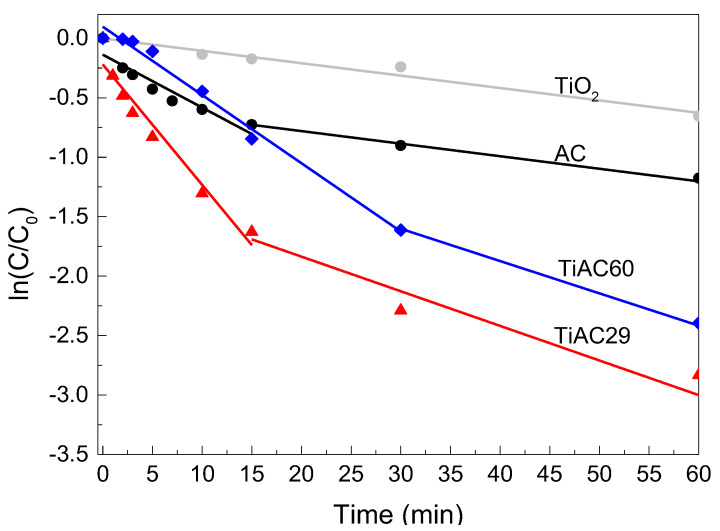
Apparent first-order kinetics of MB adsorption+photocatalytic removal for TiO_2_, AC, and TiACX catalysts under simulated visible light irradiation.

Usually, the photocatalytic degradation of dyes follows pseudo-first-order kinetics. The ln(*C*_0_/*C*) versus the *t* curves of all samples present a positive linear relationship (Table 5, Figure 12), which indicates the adsorption+photocatalytic removal of MB in these materials obeys the rules of a first-order reaction kinetics. Considering the first run, the apparent reaction rate constants of the first stage are 0.100, 0.057, 0.044, and 0.010 min^−1^ for TiAC29, TiAC60, AC, and TiO_2_, respectively. As a result, at this initial stage, the process rate for TiAC29 is 1.7, 2.3 and 10 times higher than those achieved with TiAC60, AC, and TiO_2_, respectively. These findings point out the synergetic effect of the composites, especially for sample TiAC29, when compared with the bare AC or TiO_2_.

An important factor from an economic point of view is the possibility of reusing a photocatalyst. As revealed in Table 5, after the first photocatalytic cycle, the photocatalytic activity of TiO_2_, AC, TiAC29, and TiAC60 reduced, respectively, by 27.7%, 41.6%, 61.8%, and 68.8%. This result may be due to the fact that part of MB and its partial-photodegradation products were adsorbed on the materials surface and blocked the accessibility to the active sites during the second use. Among the various regeneration methods, the thermal treatment of the catalyst is the most popular and widely used method in the industry [72]. However, the main problems with this method are the energetic requirements and low regeneration efficiency. The regeneration by solvent extraction is another process easy to implement in a real production process [73], but it cannot completely recover the full functionality of the adsorbents and has serious drawbacks associated with sustainability. As discussed in the introduction, photoregeneration is scarcely explored in the literature despite its merits and, for this reason, it was addressed here. Moreover, this strategy takes advantage of the photocatalytic response of the materials under study.

**Figure 13:**
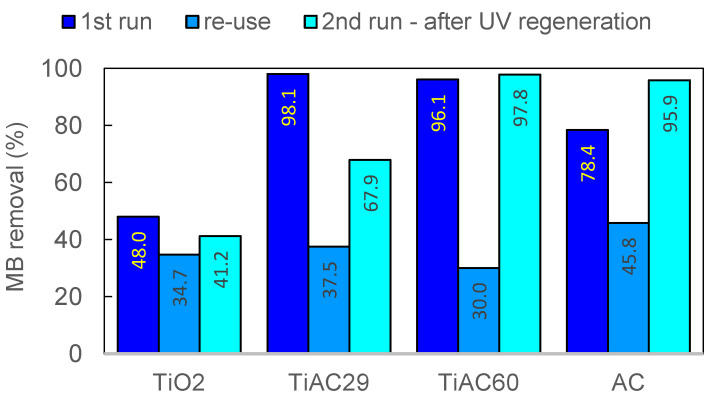
Comparison of MB removal percentage during combined adsorption + photocatalytic removal of MB, between the original (first run), re-used without regeneration (reuse) and UV-regenerated catalysts (second run after UV regeneration for 90 min).

To avoid the production of secondary pollution (for example, a solvent with MB) the pollutant adsorbed on the surface of the photocatalysts was degraded by UV light irradiation. In this regeneration experiment, the used catalysts recovered after the first run were separated, suspended in 200 cm^3^ of ultrapure water and irradiated by UV light (400 W). It was observed that the photocatalytic properties of the regenerated AC and of the regenerated composites greatly increased when compared with the reuse assay (Table 5, Figure 13). This enhancement is more noticeable for TiAC60, reaching an MB removal percentage similar to that obtained with the fresh material. In the case of AC, the UV regeneration has an even greater effect, with the regenerated material presenting a removal efficiency that is 22.3% higher than that achieved with the fresh sample. These interesting findings show that, not only is the adsorption capacity restored, but structural modifications may also occur for the AC and TiAC60 composites (e.g., extra pore network development and/or surface chemistry change), resulting in an enhanced adsorption+photocatalytic performance. This hypothesis is supported by the improved regeneration efficiency of the composite TiAC60 versus TiAC29, with the former presenting the highest amount of AC. The change in the specific surface area of AC induced by some oxidation processes under UV light irradiation is also reported in the literature [72,74]. On the other hand, according to the literature data, the photocatalytic regeneration of TiO_2_ nanotubes exhausted with MB may enhance the amount of TiO_2_ surface hydroxyl groups, thus favouring further interaction with MB and, consequently, the photocatalytic activity [75]. Nevertheless, our set of data seems to point out that the positive effect of photoregeneration is more pronounced for AC and TiAC60 than for TiAC29 (with a higher TiO_2_ loading).

#### 3.2.3. MB Removal in Real Wastewater Samples

Given the promising performance of the materials in ultrapure water containing MB, the adsorption + photocatalytic efficiency of the materials for MB removal in real wastewater was also addressed to account for a competition scenario closer to a real application.

The organic matter may block/hinder the adsorption, compete for the adsorption sites and/or increase the oxidant demand, whereas the ions may limit the electrostatic interaction [76,77,78].

For these assays, MB was dissolved in SecEf collected from an urban wastewater treatment plant. According to the spectra shown in Figure S6 (Supplementary Materials) the absorbance at 665 nm, used to quantify MB, only accounts for MB.

**Figure 14:**
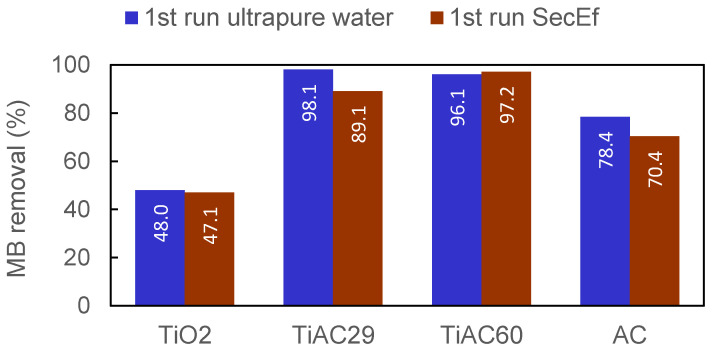
Comparison of the MB removal percentage for the assays performed in ultrapure water (first run ultrapure water) and real wastewater (first run SecEf); irradiation time = 90 min.

In the experimental conditions used (involving a well-treated and filtered SecEf), the presence of organic matter and inorganic compounds had no effect, or only led to a slight decrease (below 10 percentual points), in the combined adsorption+photocatalytic performance of the materials for MB removal (Figure 14).

**Figure 15:**
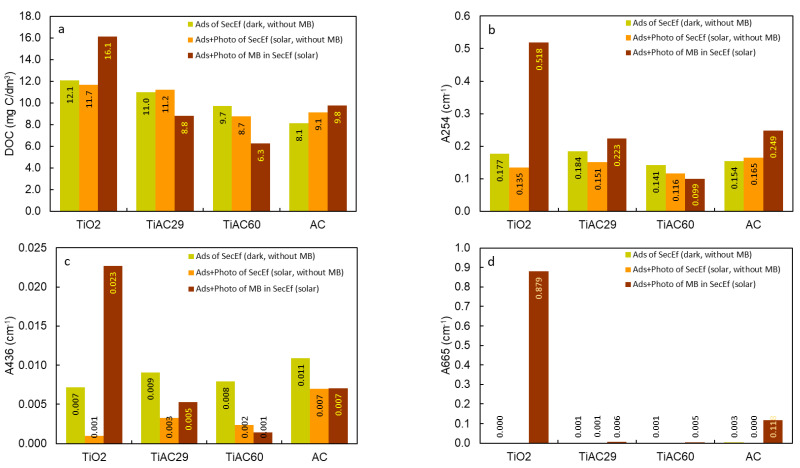
DOC (**a**), A254 (**b**), A436 (**c**) and A665 (**d**) of the SecEf after adsorption and adsorption+photocatalysis, and of the SecEf spiked with MB after adsorption+photocatalysis (25 mg/L MB and contact time = 90 min).

In a real scenario, it is also relevant to evaluate the possible advantages of the adsorption + photocatalytic process on other water parameters, such as dissolved organic carbon (DOC), and absorbance values at *λ* = 254 nm (A254) and *λ* = 436 nm (A436), corresponding, to the organic compounds containing C=C bonds (namely aromatic molecules) and colour, respectively. Firstly, the contribution of adsorption (dark conditions) and adsorption+photocatalysis (solar irradiation) to the quality of the SecEf in the absence of MB was evaluated (Figure 15). The results show that, for DOC and A254 (aromatics), there is no major improvement between the assays performed with or without solar irradiation. In contrast, for A436 (indicator of SecEf colour), the solar irradiation promoted decreases of 86%, 67%, 75%, 36% in the reactions performed with the TiO_2_, TiAC29, TiAC60 and AC, respectively, compared with the results performed without solar irradiation.

Regarding the improvement of these bulk wastewater parameters, in the case of the adsorption+photocatalytic removal of MB from SecEf under solar irradiation, the bare AC and composites present clear advantages over bare TiO_2_, with the TiAC60 composite showing, by far, the best overall performance. Interestingly, the bare AC and TiAC29 composites present a similar performance. The UV-vis absorption spectra of these solutions (Figure S6) clearly show that, after the combined adsorption+photocatalysis with composite TiAC29, the SecEf with MB present a slightly higher absorbance in the UV wavelength range compared with that of the SecEf. On the other hand, the absorbance peaks characteristic of MB (at a visible wavelength range) are no longer perceived. However, in the case of composite TiAC60, aside from the effective removal of MB, a significant removal of organic components of the SecEf is also clearly seen, as shown by the correspondent UV spectra (Figure S6).

## 4. Conclusions

In this work, novel composite photocatalysts were successfully produced by combining TiO_2_ nanoparticles and activated carbon derived from spent coffee grains, which presented solar-light induced photoactivity. The characterization results revealed the decrease in the band gap values of the composites when compared to that of the bare TiO_2_, in line with their improved combined adsorption + photocatalytic performance. Regarding the adsorption and simultaneous adsorption + photocatalytic assays performed in ultrapure water, the TiAC29 composite presented the fastest kinetic profile in the MB removal. The UV photoregeneration data revealed that TiAC60 and bare AC recovered the adsorption capacity obtained in the first cycle. When the assays were performed using a secondary effluent (SecEf) from an urban wastewater treatment plant as water matrix, all the materials led to a MB discolouration percentage similar to that obtained in the assays with ultrapure water. Moreover, the bulk water parameters (i.e., DOC, A254 and A436) clearly showed that the TiAC60 composite, besides allowing higher MB removal, also contributed to the overall improvement of the quality of SecEf. It is also important to stress that, both in the photoregeneration and SecEf-based tests, the TiAC29 performance was close to that of AC. Regardless of the assay and water matrix used, the bare TiO_2_ presented the lowest performance.

## Figures and Tables

**Table 1:** Results of chemical analyses (weight %) of the AC and TiACX materials.

Sample	%C	%N	%Ti
TiAC29	29.0	0.6	22.6
TiAC60	60.2	1.4	5.6
AC	65.2	1.5	-

**Table 2:** Textural characteristics of the materials.

Sample	*A*_BET_ (m^2^ g^−1^)	*V*_total_ ^(a)^ (cm^3^ g^−1^)	*V*_meso_ ^(b)^ (cm^3^ g^−1^)	*V*_micro_ ^(c)^ (cm^3^ g^−1^)
TiO_2_	206	0.24	0.24	0.00
AC	599	0.27	0.04	0.23
TiAC29	526	0.35	0.16	0.19
TiAC60	623	0.31	0.07	0.24

^(a)^ Evaluated at *p/p*^0^ = 0.975 in the N_2_ adsorption–desorption isotherms at −196 °C; ^(b)^ *V*_meso—_mesopore volume (pore width between 2 and 50 nm) obtained by difference between *V*_total_ and *V*_micro_; ^(c)^ *V*_micro—_micropore volume (pore width lower than 2 nm).

**Table 3:** Amounts of CO_2_, CO, and CO_2_/CO ratio on the surface of carbon-based materials determined by TPD.

Material	CO_2_ (µmol g^−1^)	CO (µmol g^−1^)	CO_2_/CO
TiAC29	972	2700	0.36
TiAC60	1848	3720	0.50
AC	2340	3612	0.65

**Table 4:** Adsorption kinetics data (*experimental conditions: q*^max^ is 45–50 mg/g, [MB] = 25.0 mg dm^−3^, *V* = 20 cm^3^, adsorption time = 90 min, *m*_adsorbent_ ~10 mg).

		1st order			2nd order			Intra-Particle			Elovich		
Sample	*q*_f_^exp (a)^	*K*_1_ ^(b)^	R^2 (c)^	*q*_f1_^calc (a)^	*K*_2_ ^(d)^	R^2 (c)^	*q*_f2_^calc (a)^	*K*_i_ ^(e)^	R^2 (c)^	*C*_i_ ^(f)^	a_e_ ^(g)^	b_e_ ^(h)^	R^2 (c)^
TiO_2_	1.29	-	-	-	-	-	-	-	-	-	-	-	-
TiAC29	37.63	3.20 × 10^−1^	0.80	24.78	2.56 × 10^−2^	1.00	37.88	25.6	1.00	0.00	2.62 × 10^32^	0.35	0.92
		5.00 × 10^−2^	1.00	7.13				3.77	0.92	21.85			
								0.71	0.89	31.39			
TiAC60	41.01	4.93 × 10^−2^	0.94	35.41	1.60 × 10^−3^	0.99	46.51	7.36	0.94	−2.64	4.77 × 10^−5^	0.10	0.99
		2.23 × 10^−2^	1.00	17.54				2.37	0.99	18.44			
AC	30.72	1.45 × 10^−2^	0.99	35.93	1.84 × 10^−3^	0.96	32.57	3.31	0.99	0.27	3.42 × 10^−1 0^	0.14	0.94

*^(^*^a*)*^*q*_f_^exp^, *q*_f1_^calc^ and *q*_f2_^calc^ are the amounts of adsorbed MB per gram of adsorbent (mg g^−1^) after 90 min of contact time obtained from experiment and calculated for first- and second-order kinetics; ^(b)^ *K*_1_ is the pseudo-first-order rate constant of MB sorption (min^−1^); ^(c)^ R^2^ is the correlation coefficient; ^(d)^ *K*_2_ is the pseudo-second-order adsorption rate (mg g^−1^ min^−1^); ^(e)^ *K*_i_ is the intra-particle diffusion rate constant (mg g^−1^ min^−0.5^); ^(f)^ *C*_i_ is the thickness of the boundary layer; ^(g)^ a_e_ is the initial adsorption rate constant (mg g^−1^ min^−1^); ^(h)^ b*_e_*is related to the extent of surface coverage and the activation energy for chemisorption (g mg^−1^).

**Table 5:** Kinetic data from combined adsorption+photocatalytic MB removal from ultra-pure water ^(a)^.

Photocatalyst	Run	%R ^(b)^	*K*_11_ ^(c)^ (min^−1^)	R^2 (d)^	*K*_12_ ^(c)^ (min^−1^)	R^2 (d)^
TiO_2_	1	48.0	1.0 × 10^−2^	0.97		
	2 ^(e)^	34.7	2.3 × 10^−3^	0.89		
	2 ^(f)^	41.2	3.8 × 10^−2^	0.82	2.3 × 10^−3^	0.99
TiAC29	1	98.1	1.0 × 10^−1^	0.95	2.9 × 10^−2^	0.98
	2^(e)^	37.5	1.2 × 10^−2^	0.84	5.0 × 10^−4^	0.68
	2 ^(f)^	67.9	5.2 × 10^−2^	1.00	3.7 × 10^−3^	0.97
TiAC60	1	96.1	5.7 × 10^−2^	0.99	2.7 × 10^−2^	0.99
	2^(e)^	30.0	1.4 × 10^−2^	0.77	7.0 × 10^−4^	0.94
	2 ^(f)^	97.8	4.4 × 10^−2^	0.94	1.4 × 10^−3^	0.99
AC	1	78.4	4.4 × 10^−2^	0.89	1.1 × 10^−2^	1.00
	2 ^(e)^	45.8	8.2 × 10^−3^	0.80	1.0 × 10^−3^	0.66
	2 ^(f)^	95.9	6.2 × 10^−2^	0.87	6.0 × 10^−4^	0.93

^(a)^ Operating conditions: catalyst dosage = 0.5 g dm^−3^, V = 0.02 dm^3^, [MB] = 25 mg dm^−3^, contact time was 90 min except TiO_2_ where contact time was shorter (t = 60 min); ^(b)^ Discolouration percentage (adsorption+photocatalysis) calculated from: ((*A*_0_ − *A*_t_)/*A*_0_) × 100%, where *A*_0_ and *A*_t_ are the absorption of MB solution at *t* = 0 and contact time (min), respectively; ^(c)^ *K*_11_ and *K*_12_ are first-order kinetic rate constants for the first (*K*_11_) and second (*K*_12_) stage, respectively, calculated from ln(*C*/*C*_0_) = −*Kt*, where *C*_0_ and *C* (mg dm^−3^) are the concentrations of MB at *t* = 0 and time *t* (min), respectively, and *K* (min^−1^ ) is the rate constant; ^(d)^ R^2^ is the correlation coefficient; ^(e)^ second run normalized to 10 mg of the photocatalyst; ^(f)^ second run of regenerated catalyst by UV light irradiation (normalized values).

## Supplementary Materials

The following are available online at https://www.mdpi.com/article/10.3390/nano11113016/s1, Figure S1: Photodegradation of MB solution under simulated solar light and using TiO_2_ no thermal treated, as well as calcinated at 150 °C, 200 °C and 250 °C as catalysts. Conditions: [MB] = 25 mg/L, [Catalyst] = 0.5 g/L and VMB = 20 mL, Figure S2: TEM image of TiO_2_ sample, Figure S3: XPS survey spectra of AC taken from three different sample zones Figure S4: Fitting with Elovich model to the MB adsorption onto TiACX systems and AC, Figure S5: Removal of MB solution by onto bare materials and composites under simulated solar light irradiation, Figure S6: Spectra of the SecEf and MB solutions in SecEf after combined adsorption+photocatalysis of SecEf under solar irradiation ([MB] = 25 mg/dm^3^ and contact time = 90 min).
