# Supplementary material for: Solar Light-Induced Methylene Blue Removal over TiO2/AC Composites and Photocatalytic Regeneration
Source: Nanomaterials (Basel). 2021 Nov 10;11(11):3016. doi: 10.3390/nano11113016 (PMC8625254; doi:10.3390/nano11113016)
Supplement: Supplementary file 1 [file nanomaterials-11-03016-s001.zip › nanomaterials-1437758-supp materials.pdf]

nanomaterials

MDPI

Supplementary Materials

Solar Light-Induced Methylene Blue Removal over TiO_2/AC Composites and Photocatalytic Regeneration

Fernanda Dalto [1,†], Iwona Kuźniarska-Biernacka [1,†], Clara Pereira [1], Elsa Mesquita [2], Olívia Salomé G. P. Soares [3], M. Fernando R. Pereira [3], Maria João Rosa [2], Ana S. Mestre [4,*], Ana P. Carvalho [4] and Cristina Freire [1,*]

[1] REQUIMTE/LAQV, Departamento de Química e Bioquímica, Faculdade de Ciências, Universidade do Porto, Rua do Campo Alegre s/n, 4169-007 Porto, Portugal; up201309985@fc.up.pt (F.D.); iwonakb@fc.up.pt (I.K.-B.); clara.pereira@fc.up.pt (C.P.), acfreire@fc.up.pt (C.F.)
[2] Water Quality and Treatment Laboratory, Urban Water Unit, Hydraulics and Environment Department, LNEC—National Laboratory for Civil Engineering, Av. Brasil 101, 1700-066 Lisboa, Portugal; emesquita@lnec.pt (E.M.); mjrosa@lnec.pt (M.J.R.)
[3] LSRE-LCM, Departamento de Engenharia Química, Faculdade de Engenharia, Universidade do Porto, Rua Dr. Roberto Frias s/n, 4200-465 Porto, Portugal; osgps@fe.up.pt (O.S.G.P.S.); fpereira@fe.up.pt (M.F.R.P.)
[4] Centro de Química Estrutural, Faculdade de Ciências, Universidade de *Lisboa,* Campo Grande, 1749-016 *Lisboa,* Portugal; ana.carvalho@fc.ul.pt (A.P.C), asmestre@fc.ul.pt (A.S.M.)
* Correspondence: asmestre@fc.ul.pt (A.S.M.); acfreire@fc.up.pt (C.F.)
† These authors contributed equally to this work.

Adsorption Kinetic Models

The following equation given by Lagergren describes a pseudo-first order model [1]:

$$\log(q_e - q) = \log q_e - k_1 t/2.303 \qquad \text{(S1)}$$

In this equation, q_e and q are the amounts of MB adsorbed (mg g^{-1}) at the equilibrium time and time t (min) respectively, and k_1 is the rate constant of sorption (min^{-1}). The rate constant and the corresponding linear regression correlation coefficient values were determined from the straight line plots of log (q_e–q) versus t.

The McKay and Ho, pseudo-second order model [2] based on the equilibrium sorption is expressed by the following equation:

$$\frac{t}{q} = \frac{1}{k_2 q_e^2} + \frac{t}{q_e} \qquad \text{(S2)}$$

where q_e and q represent the amount of MB adsorbed (mg g^{-1}) at the equilibrium and at any time t (min), respectively, and k_2 is the pseudo-second order rate constant (g mg^{-1} min^{-1}). The values of q_e and k_2 were determined from the straight-line plots of t/q versus t.

The intra-particle diffusion (Weber-Morris) model [3] is represented by the following equation:

$$q = k_{id}\sqrt{t} + C_i \qquad \text{(S3)}$$

where k_{id} is the intra-particle diffusion rate constant (mg g^{-1} min$^{-0.5}$).

Based on this model, when the intra-particle diffusion takes part in the sorption process, the plot of q against $\sqrt{t}$ is linear. The value of the intercept (C) gives an idea about the thickness of the boundary layer i.e., the larger the intercept the higher the boundary layer effect.

The linear form of Elovich equation [4,5] is given as:

$$q = \frac{\ln(a_e b_e)}{b_e} + \frac{1}{b_e} lnt \qquad \text{(S4)}$$

where a_e represents the initial sorption rate constant (mg g^{-1} min^{-1}) and b_e is related to the extent of surface coverage and the activation energy for chemisorption (g mg^{-1}). The values of a_e and b_e can be computed from the plot of q versus ln t.

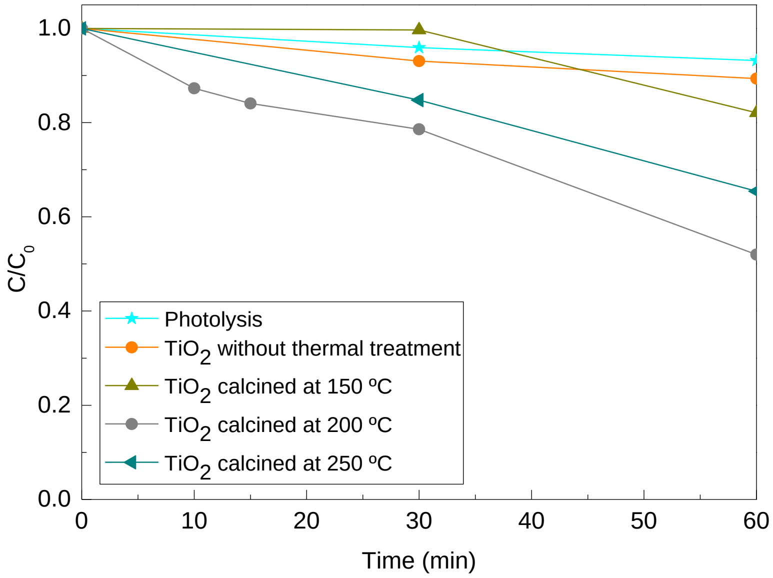

Figure S1. Photodegradation of MB solution under simulated solar light and using TiO_2 not thermally treated, as well as calcined at 150 °C, 200 °C and 250 °C as catalysts. Conditions: [MB]=25 mg/L, [Catalyst] = 0.5 g/L and V_{MB} = 20 mL.

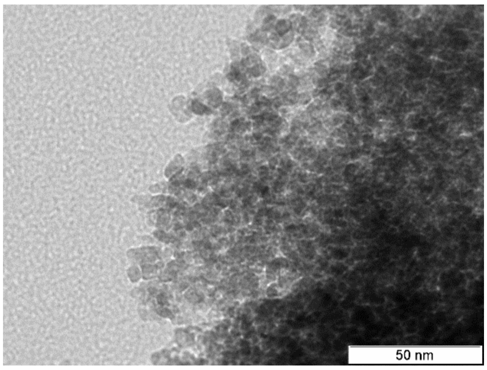

Figure S2. TEM image of TiO_2 sample treated at 200 °C.

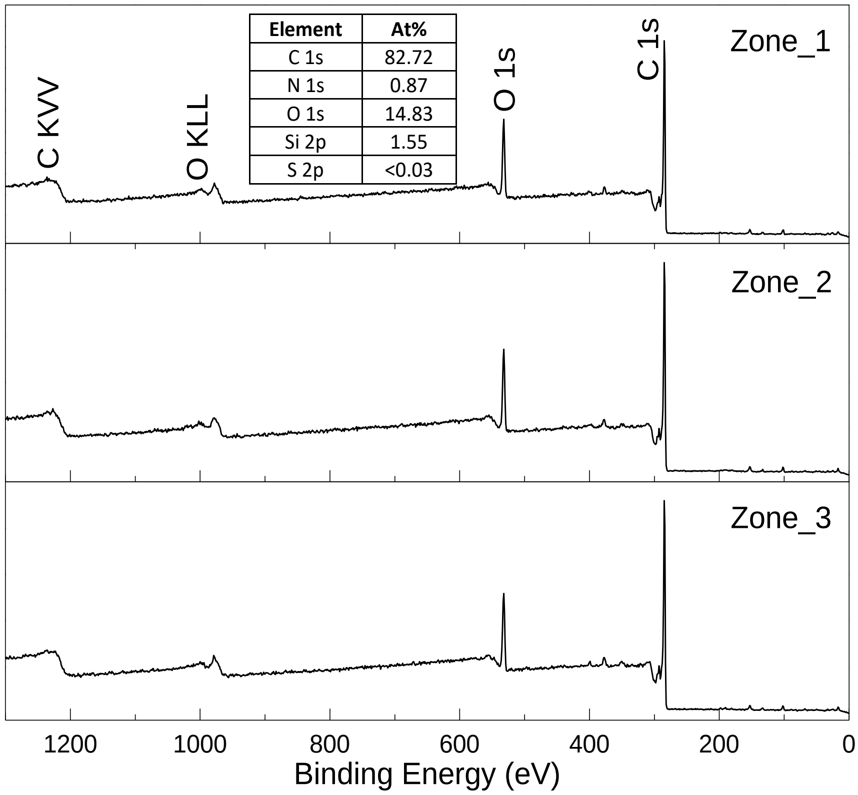

Figure S3. XPS survey spectra of AC taken from three different sample zones.

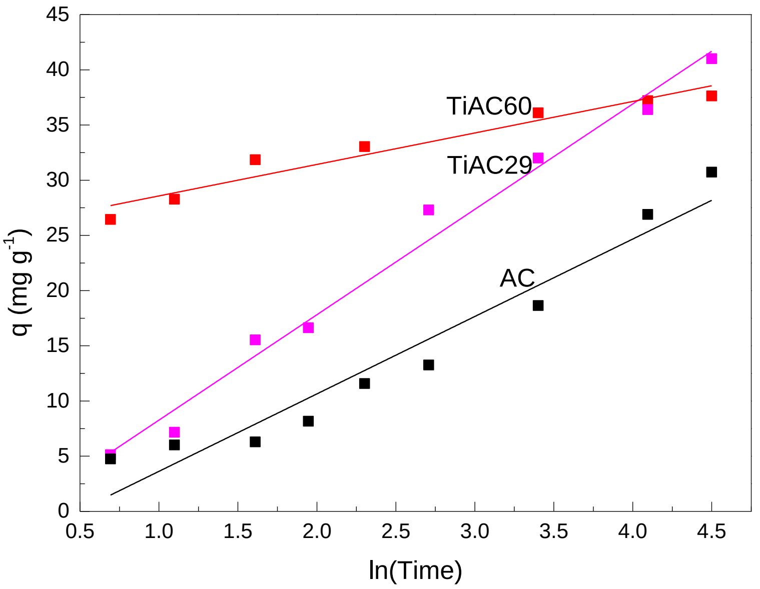

Figure S4. Fitting with Elovich model to the MB adsorption onto TiACX systems and AC.

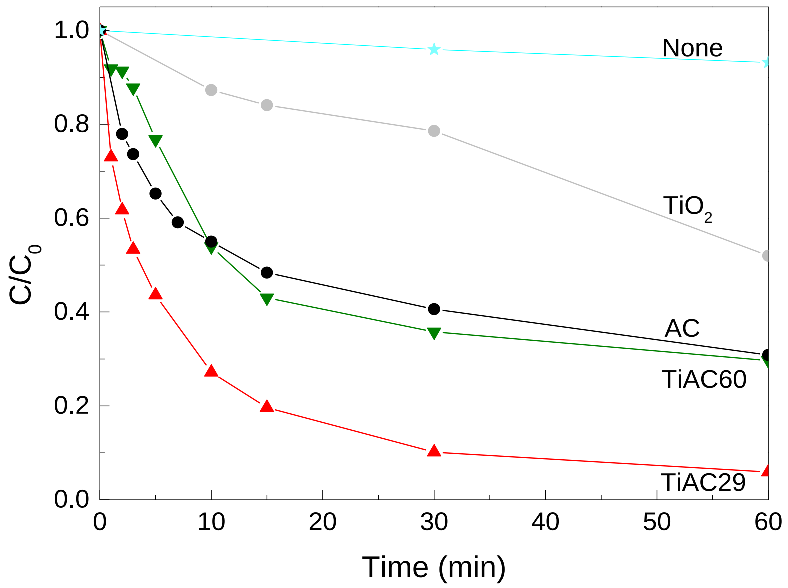

Figure S5. Removal of MB solution by onto bare materials and composites under simulated solar light irradiation.

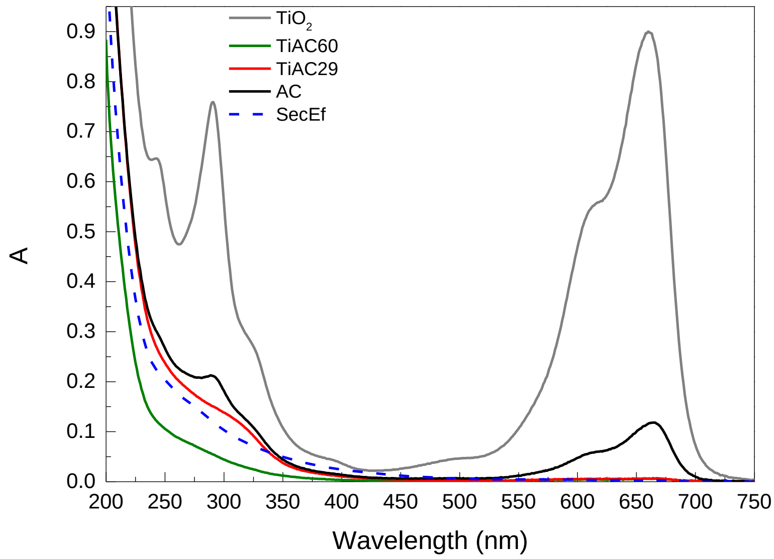

Figure S6. Spectra of the SecEf and MB solutions in SecEf after combined adsorption+photocatalysis of SecEf under solar irradiation ([MB] = 25 mg/dm^3 and contact time = 90 min).

References

1. Lagergren, S. Handlingar. *Band* **1898,** *24*, 1–39.
2. Ho, Y.S.; McKay, G. Pseudo-second order model for sorption processes. *Process Biochem.* **1999,** *34*, 451–465.
3. Cheung, W.H.; Szeto, Y.S.; McKay, G. Intraparticle diffusion processes during acid dye adsorption onto chitosan. *Bioresour. Technol.* **2007,** *98*, 2897–2904.
4. Juang, R.-S.; Chen, M.-L. Application of the Elovich Equation to the Kinetics of Metal Sorption with Solvent-Impregnated Resins. *Ind. Eng. Chem. Res.* **1997,** *36*, 813–820.
5. Zhang, J.; Stanforth, R. Slow Adsorption Reaction between Arsenic Species and Goethite (α-FeOOH): Diffusion or Heterogeneous Surface Reaction Control. *Langmuir* **2005,** *21*, 2895–2901.
